# Scaling a waterfall: a meta-ethnography of adolescent progression through the stages of HIV care in sub-Saharan Africa

**DOI:** 10.7448/IAS.20.1.21922

**Published:** 2017-09-15

**Authors:** Shannon Williams, Jenny Renju, Ludovica Ghilardi, Alison Wringe

**Affiliations:** ^a^ Department of Population Health, London School of Hygiene and Tropical Medicine, London, UK; ^b^ Department of Epidemiology and Biostatistics, Kilimanjaro Christian Medical University College, Moshi, Tanzania

**Keywords:** adolescents, HIV /AIDS, sub-Saharan Africa, antiretroviral therapy, HIV care cascade, HIV care continuum, qualitative research, meta-ethnography, stigma

## Abstract

**Introduction**: Observational studies have shown considerable attrition among adolescents living with HIV across the “cascade” of HIV care in sub-Saharan Africa, leading to higher mortality rates compared to HIV-infected adults or children. We synthesized evidence from qualitative studies on factors that promote or undermine engagement with HIV services among adolescents living with HIV in sub-Saharan Africa.

**Methods**: We systematically searched five databases for studies published between 2005 and 2016 that met pre-defined inclusion criteria. We used a meta-ethnographic approach to identify first, second and third order constructs from eligible studies, and applied a socio-ecological framework to situate our results across different levels of influence, and in relation to each stage of the HIV cascade.

**Results and discussion**: We identified 3089 citations, of which 24 articles were eligible for inclusion. Of these, 17 were from Southern Africa while 11 were from Eastern Africa. 6 explored issues related to HIV testing, 11 explored treatment adherence, and 7 covered multiple stages of the cascade. Twelve third-order constructs emerged to explain adolescents’ engagement in HIV care. Stigma was the most salient factor impeding adolescents’ interactions with HIV care over the past decade. Self-efficacy to adapt to life with HIV and support from family or social networks were critical enablers supporting uptake and retention in HIV care and treatment programmes. Provision of adolescent-friendly services and health systems issues, such as the availability of efficient, confidential and comfortable services, were also reported to drive sustained care engagement. Individual-level factors, including past illness experiences, identifying mechanisms to manage pill-taking in social situations, financial (in)stability and the presence/absence of future aspirations also shaped adolescents HIV care engagement.

**Conclusions**: Adolescents’ initial and ongoing use of HIV care was frequently undermined by individual-level issues; although family, community and health systems factors played important roles. Interventions should prioritise addressing psychosocial issues among adolescents to promote individual-level engagement with HIV care, and ultimately reduce mortality. Further research should explore issues relating to care linkage and ART initiation in different settings, particularly as “test and treat” policies are scaled up.

## Introduction

Globally, it has been estimated that approximately 2.1 million adolescents aged 10–19 years are living with HIV, with nearly 85% of this population residing in sub-Saharan Africa (SSA) [1]. Whilst initiatives to improve access to antiretroviral therapy (ART) in low- and middle-income countries (LMIC) contributed to a 30% decline in AIDS-related mortality between 2005 and 2012 [2], adolescents have been left behind. In the same time-period, AIDS-related mortality amongst adolescents increased by 50%, with over 90% of these deaths occurring in SSA [1].

The clustering of adolescent AIDS-related mortality in SSA is largely attributable to an approximate 10-year time-lapse in ART interventions compared to high-income nations [3]. Many of the children perinatally infected in SSA, while prevention of mother-to-child transmission (PMTCT) programme coverage was still low, are now reaching their adolescence [4,5]. However, less than 30% of adolescent girls and 10% of adolescent boys aged 15–19 years in SSA report ever having undergone an HIV test [6]. In addition, the regional adolescent population has been projected to double by 2050, with adolescents making up approximately 46% of the total population [6].

Recent global initiatives have called for adolescent health to be prioritized as young people represent a “triple dividend” investment – of benefit now, to the next generation of adults and to the health of generations to come [7]. At the heart of these initiatives lies the need to prevent and treat infections among this population, making care and treatment for ALHIV a policy priority for many African governments. This places new pressures on health systems and services to meet the needs of a rapidly expanding generation of both diagnosed and undiagnosed adolescents living with HIV (ALHIV) [5,8].

Transition from childhood into adulthood is characterized by rapid physical, mental and sexual development, and in addition to this, ALHIV have complex emotional, psychological and health-related requirements that need to be reconciled to effectively manage their lives with HIV [4]. In SSA, the majority of ALHIV under 14 years of age have been infected perinatally [9] and many are orphaned by one or both parents [10] forcing them into early emotional maturity and adult responsibilities. Furthermore, while some have been on ART since a very young age, others have been living with the virus untreated for over a decade. Without access to ART, those untreated are not expected to survive beyond their teenage years [9]. For those that do survive, great efforts are needed to increase care access and improve retention on ART to reduce AIDS-related mortality and onward horizontal transmission of HIV [9].

Quantitative research indicates that in many settings ALHIV experience higher rates of loss to follow-up and virological failure than both adults and children living with HIV [11–14]. Geographically discreet qualitative studies in different countries in SSA suggest that stigma, challenges in disclosure and consent policies for testing and treatment are reasons for faltering ALHIV care engagement, however, there has been no published synthesis of these studies. Meta-ethnographic methods enable an in-depth assessment of various health-related research topics [15,16], including factors that influence patient care experiences and medication adherence in LMIC [17,18]. Meta-ethnography aims to expand upon, or develop new interpretations of qualitative data. The “constant comparison” approach is used to determine if themes across varying contexts are reciprocal (complementary concepts) or refutational (contradictory concepts), supporting further hypothesis development [17,19–21]. Our meta-ethnography includes qualitative studies undertaken across the region, in order to understand the most influential issues affecting adolescent initiation of, and retention in, HIV care in SSA. Our synthesis of 10 years of qualitative research conducted since the advent of ART expansion enables a longitudinal line of inquiry, allowing us to assess changes across time, in different countries in SSA and across all stages of the HIV care cascade.

### Theoretical frameworks

The HIV care cascade describes progression through HIV diagnosis, care and treatment services and is commonly defined in five stages: (i) HIV diagnosis, (ii) linkage to care, (iii) engagement/retention in care, (iv) ART initiation and (v) viral suppression [22, 23]. The cascade has been widely used as a framework to explore reasons for patient attrition between these five stages, with some variations in the definitions for each stage depending on the study design or nature of the data that are available. For example, in relation to stage v, qualitative studies often explore patients’ experiences of adherence to antiretroviral therapy, rather than investigating viral suppression per se, which is more commonly measured in studies with a quantitative design [24,25].

For adolescents, traversing the cascade is complicated by the lack of autonomy in care-seeking that characterizes this age-group, including the requirement for guardian accompaniment to access services. Further disruptions in HIV care among adolescents have been noted to occur during the transition from paediatric into adult care [26] or as they transition in and out of PMTCT programmes [27,28]. Such challenges perpetuate the risk of attrition and associated mortality [26,29]. “Engagement” with the HIV care cascade involves the willingness and ability of ALHIV to undergo HIV testing, initiate treatment, adhere to ART and negotiate the linkages between different HIV services and models of care (e.g. paediatric and adult; pre-ART and ART). This engagement with HIV care and treatment is influenced by a multitude of factors including basic service access, economic capability, and awareness of their HIV status [30].

**Figure 1.**
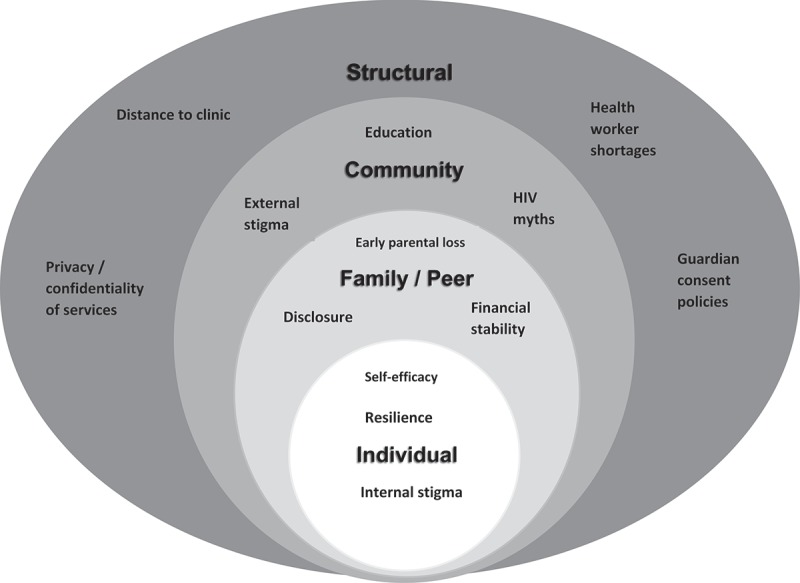
The four levels of the socio-ecological model that have the potential to affect health behaviours, with examples of factors that operate at each level.

The socio-ecological model (SEM) has been frequently used across HIV literature to illustrate the range of social influences on health behaviours, including care fallout [31,32]. The SEM is particularly useful in exploring health decisions made by individuals in LMIC or communities with high levels of disease stigmatization [33–35]. The model illustrates four levels of influence on health decisions (Figure 1): i) s*tructural* factors, such as health systems, policies and underlying poverty; ii) *community* factors, including social norms around treatment-seeking and beliefs surrounding disease aetiology; iii) *family and peer* influences, including expectations of behaviours within these social contexts; iv) and *individual* factors at the model’s core, including knowledge, perceptions, self-efficacy and risks associated with care-seeking. Influential factors may act across more than one level of the SEM: for example, family influences on adolescents’ care-seeking behaviours may be affected by structural factors such as economic circumstances of the household or consent policies that limit access to services.

## Methods

### Search strategy

For each search, five essential concepts were identified: HIV, adolescents, SSA, barriers /facilitators and each of the five stages of the care cascade. All possible search terms and synonyms for each concept were established and multiple spellings were incorporated. We searched five databases known for having a high volume of social and health-related research or articles specific to adolescents: PubMed, Web of Science, Scopus, Global Health and ADOLEC. Titles and abstracts of identified literature were imported into EndNote and reviewed to eliminate duplicates. References from highly relevant articles and systematic reviews were further checked for additional eligible articles.

### Inclusion /exclusion criteria

Studies were included if they involved qualitative or mixed methods, were published between January 2005 and March 2016, included an adolescent population in the age range 10–19 years (if the study included children, young adults or adults, it was included so long as the adolescent population was distinct in the results), were conducted in one or more countries within SSA (or included a defined population in SSA) and stated aims that included an assessment of factors influencing adolescent engagement with at least one stage of the HIV care cascade. Only articles published in English were included.

### Quality appraisal

The importance of appraising the quality of health research is increasingly recognized, although challenges include the lack of a standardized method or approach [36,37]. The Oxford Critical Appraisal Skills Programme (CASP) criteria is one of the most widely used checklists for qualitative research, enabling an assessment to be made of a study’s validity, rigour and relevance [38].

Two researchers (SW, LG) reviewed all articles according to the ten-point scale proposed by CASP and scored them independently. One point was given for each checklist criterion present in the research article and zero points were given for absent criterion. Individual scores were compared and differences deliberated until an agreement was reached. Any scoring not reached in agreement was submitted to a third researcher (AW) for final review.

### Thematic analysis

Thematic constructs were noted according to the methods of conducting meta-ethnographic analysis suggested by Noblit and Hare [19] and findings were applied to the SEM. In each article, first-order constructs were identified through study participant quotations in the text. Second-order constructs were then distinguished from these based on the researchers’ perspectives, mainly presented in the discussion sections of research articles. Comparison of first and second order constructs was examined chronologically across studies that explored the same stage of HIV care. Articles that covered more than one stage of care were catalogued into each stage of the cascade sequentially, for a complete analysis of themes specific to that stage. Third order constructs were then defined to contextualize all identified themes across the entire cascade of care, noting reciprocal and highly salient constructs.

## Results

### Characteristics of included studies

**Figure 2.**
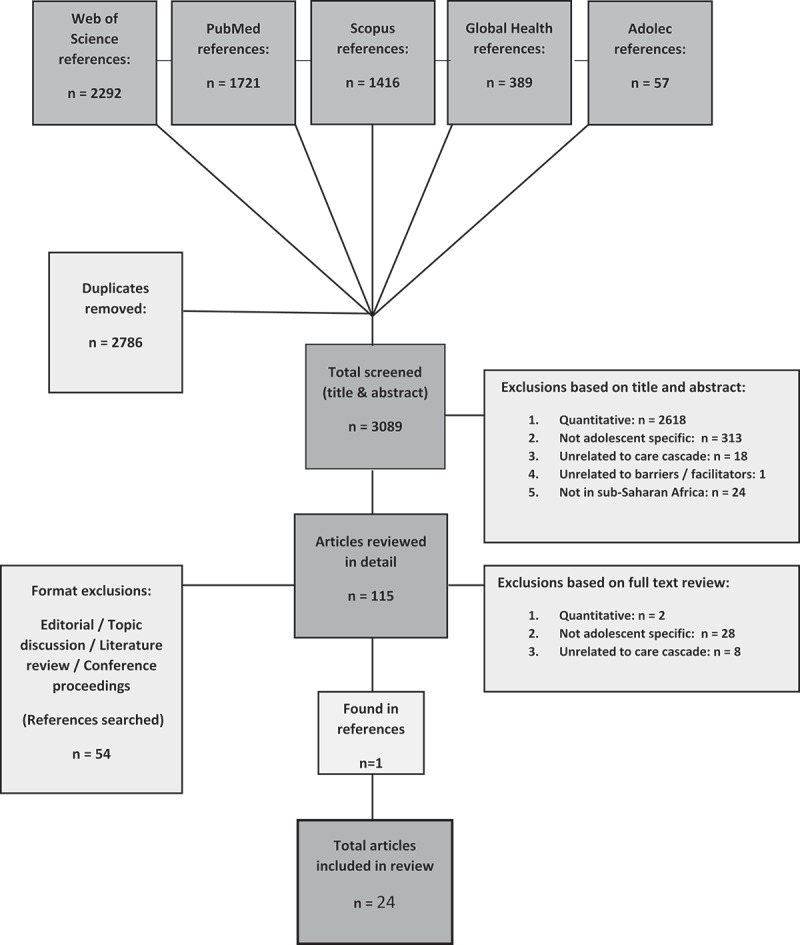
Flow diagram showing search strategy and number of articles identified at each stage.

In total, 3089 publications were identified through the search. Following the review of titles and abstracts, 2974 were excluded for not meeting the inclusion criteria, most of which were quantitative studies. The remaining 115 articles were reviewed in full, following which an additional 92 were excluded (the majority for lacking results specific to adolescents). 1 additional relevant study was discovered searching article references. In total, 24 qualitative and mixed methods articles were selected for inclusion in the meta-ethnography (Figure 2).

The eligible articles were catalogued in accordance to HIV care cascade stages (i.e. HIV diagnosis, linkage to care, engagement/retention in pre-ART care, ART initiation and adherence), and within those stages arranged chronologically by year of research. Table 1 presents the characteristics of included studies.

**Table 1.** Characteristics of included qualitative and mixed methods studies

#	Care stage(s)	1st author, year	Year of research	Location	Setting	Age range of adolescents	Data collection method	Aims
1	1	Rassjo, 2007	2004	Uganda	Urban slums; Kampala	10–21^a^	FGD and IDI: adolescents & young adults.	To evaluate young people’s perceptions regarding VCT and the barriers and facilitators to testing.
2	1	Izugbara, 2009	2004	Uganda, Malawi	Urban and rural	14–19	FGD: adolescent males	To offer youth perspectives on VCT; specifically, how young men navigate their identities and masculinity as they relate with HIV services.
3	1	Ferrand, 2011	2009	Zimbabwe	Clinics; urban suburbs, Harare	10–18	IDI: adolescents and guardians.^b^	To investigate adolescent & guardian acceptability and rate of service uptake of PITC, as well as risk perceptions of MTCT.
4	1	Francis, 2010	2009	South Africa	Industrial zones; Shongweni, Durban	16–19	IDI: out-of-school youth.	To collect out-of-school/working youth perceptions on VCT and survey knowledge about HIV using peer researchers.
5	1	Ntsepe, 2014	2011	South Africa	Urban community venues & schools: Cape Town, Durban	12-adult ^a^	FGD: adolescents, young people & adults.	To evaluate differences in perception, acceptability and uptake of HTC in different racial communities in South Africa.
6	1	Strauss, 2015	2012–2013	South Africa	Rural schools: Vulindlela, KwaZulu-Natal	>16 secondary school students	FGD: in-school adolescents.	To examine barriers and facilitators to HCT uptake offered to young people in school settings.
7	5	Schenk, 2014	2007	Kenya	Areas within 5 km of paediatric HIV clinics in Narobi, Eastern & Nyanza provinces	<15 ^a^	IDI: Caregivers, HCW, clinic managers & MoH reps.	To define demand side barriers to uptake of HIV services for perinatally infected children <15, with an elaboration on the challenges specific to adolescents.
8	5	Mavhu, 2013	2009	Zimbabwe	Africaid community support groups – Harare (urban)	15–18	FGD & IDI: ALHIV, caregivers, HCW. Life narratives: ALHIV.^b^	To strengthen the evidence base for a psychosocial intervention planned by Africaid for ALHIV utilizing community spaces to host peer support groups.
9	5	Lowenthal, 2014	2009–2010	Botswana	Clinics: Gaborone (urban)	10–19	IDI: ALHIV	To identify the culturally specific factors necessary to adapt in order to best utilize western tools for psychosocial assessment when exploring correlates of adherence for ALHIV in SSA.
10	5	Li, 2010	2010	South Africa	Urban clinic: Cape Town	10–15	FGD: ALHIV	To identify experiences, needs and illness perspectives of both behaviourally and perinatally infected ALHIV.
11	5	Denison, 2015	2011–2012	Zambia	Two ART clinics (1 children’s, 1 general) – Ndola (urban)	15–18	IDI: ALHIV and caregivers.	To explore ART adherence from the perspective and experiences of older adolescents (15–18) and their caregivers.
12	5	Nyogea, 2015	2011–2012	Tanzania	Chronic disease clinic – Ifakara (rural)	13–17 Qualitative (equal component)	FGD, IDI: ALHIV & caregivers.^b^	To estimate adherence levels and find the determinants of ART adherence for children and adolescents.
13	5	Vreeman, 2012	2012	Kenya	AMPATH clinics: urban & rural	10–18	IDI: adolescents & caregivers. Cognitive interviewing: ALHIV.	To improve understandability of paediatric ARV adherence measurement items used in resource limited settings through cognitive interviewing.
14	5	Mutumba, 2015	2012	Uganda	Urban clinics	13–19	IDI: ALHIV	To identify the psychosocial challenges and coping strategies of perinatally infected ALHIV.
15	5	Mupambireyi, 2014	2011–2013	Zimbabwe	Low income, suburbs	11–13	FGD & IDI: adolescents & caregivers.^b^	To explore peer social support experiences of young, perinatally infected adolescents.
16	5	Bernays, 2015	2011–2013	Uganda, Zimbabwe	Clinics: Multi-site, recruited from ARROW Trial	11–13	IDI: ALHIV	To examine experiences of living with HIV on ART from the perspective of young ALHIV, carers and HCWs.
17	5	Cluver, 2015	2013	South Africa	Clinics: Eastern Cape, low resource province	10–19	IDI: ALHIV. FGD: adolescents, caregivers & parents.^b^	To examine associations between knowledge of HIV+ status (disclosure) and ART adherence.
18	2, 5	Kunapareddy, 2014	2007	Kenya	AMPATH clinics: urban & rural	10–16	FGD & IDI: ALHIV	To identify key factors related to medication adherence for perinatally infected ALHIV on ART culturally specific to SSA.
19	2, 5	Mattes, 2014	2008–2011	Tanzania	Orphanages and private homes – Tanga	9–19 ^a^	IDI: ALHIV & caregivers. Observation: ALHIV. Photo elicitation: ALHIV.	To compare national guidelines for HIV disclosure and treatment management with the lived realities of ALHIV.
20	2,3	Petersen, 2010	2010	South Africa	Hospital – Durban (rural)	14–16	IDI: ALHIV and caregivers.	To develop an understanding of psycho-social challenges and protective influences promoting socio-emotional coping; to inform mental health promotion and HIV prevention programmes.
21	2, 5	Hodgson, 2012	2010	Zambia	Clinics: 1 Kalomo province (rural); 2 Lusaka & Kitwe province (urban)	10–19	IDI & FGD: adolescents, parents & guardians.	To explore and document the educational, psychosocial and SRH needs of ALHIV.
22	2, 3, 4, 5	Mburu, 2014	2010	Zambia	HIV clinics: Kalomo (rural), Kitwe & Lusaka (urban)	10–19	IDI & FGD: adolescents, parents & caregivers.	To examine the experiences of ALHIV, with emphasis on how factors fit within the construct of the social ecosystem.
23	3, 4,5	Midtbo, 2012	2011	Botswana, Tanzania	Botswana: 1 urban hospice, 1 rural clinic. Tanzania: urban community support setting.	12–20^a^	IDI, FGD & observation: ALHIV & young people	To understand and identify pathways between disclosure, ART and psychosocial wellbeing, from perspectives of ALHIV.
24	2, 3, 4	Busza, 2014	2011	Tanzania	Urban and rural community settings: Dar es Salaam	15–19	FGD: HBC providers. IDI: ALHIV, caregivers & HBC providers.	To examine the experiences of ALHIV in order to identify ways to improve HBC to better meet their needs.

^a^ Adolescent population distinct in results; ^b^ Study was mixed methods

Care cascade stages: **1** – testing; **2** – care initiation; **3** – in care, awaiting ART; **4** – ART initiation; **5** – on ART, working towards/maintaining viral suppression

**Figure 3.**
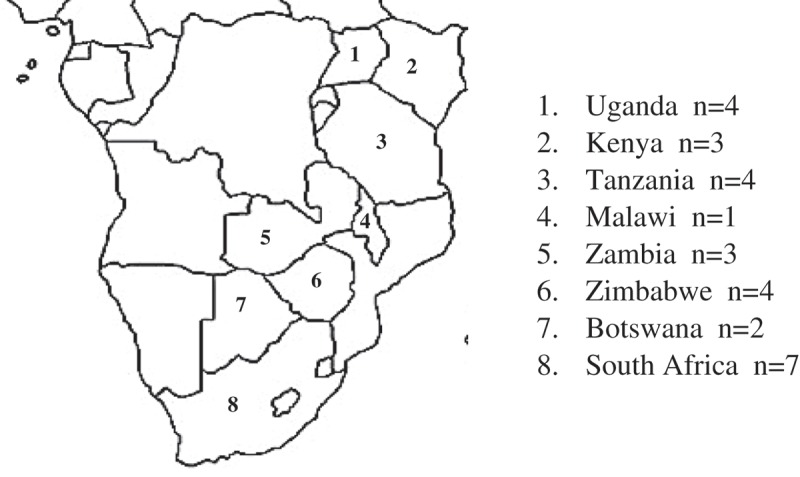
Map of countries in SSA with published qualitative research on the ALHIV care cascade.

The majority of identified studies were conducted in Southern and Eastern Africa with notable gaps in some high prevalence countries, including Mozambique, Swaziland and Lesotho (Figure 3).

Out of the 24 studies included in our review, 6 explored issues related to cascade stage one, HIV testing, and 11 explored factors related to stage five, ART adherence. The remaining 7 studies examined multiple stages of the cascade. No qualitative studies were identified that specifically aimed to explore factors influencing ALHIV linkage to care following diagnosis (stage 2), engagement/retention in general medical care (stage 3) or initiation onto ART (stage 4).

### Quality assessment

Eighteen out of 24 (75%) of the included studies were rated moderate to high quality, scoring 7–10 points on the CASP scale; and none of the studies scored below six points. The CASP criteria least achieved (absent in 13 out of 24 articles (54%)) was a failure to elaborate on the relationship between researchers and study participants (CASP criteria 6).

### Themes influencing the engagement of ALHIV in care and treatment services

Twelve third-order themes were identified, many of which acted across multiple levels of the SEM (Table 2). Salient themes are presented by order of importance, determined by number of studies they were reported in and instances of overlap within the models.

**Table 2.** Themes: First-, second- and third-order constructs and their positioning across the levels of the SEM

3rd order constructs	2nd order constructs + source papers	1st order constructs + source papers	SEM LEVELS	Care stages
Stigma *n* = 23	Anticipated stigma surrounding being seen utilizing HIV services (1,2,3,4,5,6,17,20)	*“Let me say there is this particular lady who works at the clinic who is also a neighbour, this lady might see that particular person going to do a HIV test and tell the family…” – Strauss, p 7.*		
	Anticipation of knowing people at the clinic (4,6)			
	Anticipated isolation from peer group (1,2,4,5)	*“…if you are going for an HIV test its going to be stigmatized that you are sexually active and a lot of us youngsters do not want people to think that and know that.” –* Ntsepe, p 144.		
	Anticipated assumptions regarding sexual activity (2,3,5,21)			
	Anticipation of unwanted disclosure after being seen using HIV services (20,24)	*“My child was pinned a red label on his shirt by his teacher after he knew the HIV and treatment status of the child.”* – Nyogea, p 7.	I,F,C,S	All
	Anticipated stigma from family member (2,3,5,12,14,18)			
	Enacted stigma from teachers towards ALHIV (7,12,14,22,23)			
	Anticipation of disclosure at school by carrying medication (11,14)			
	Desire to appear “normal”, avoid enacted stigma associate with symptoms (14,16)	*“Nurses in Soweto do not treat you like human beings. They stigmatize you and by the time you leave there you have a different mindset”* – Forrest, p S58.		
	Enacted stigma from judgemental or reprimanding HCWs (13,15,18,19,21)			
	Anticipated stigma prioritizes secrecy routines over medication adherence (7,8,9,10,11,12,13,14,16,18,23)			
Self-efficacy surrounding adaptive mechanisms *n* = 19	Fear of being incapable of making lifestyle changes (1,2,5,6)	*“It isn’t just to go and test, you feel everything, you never know – because they say a piece of paper can upset your life.” –* Ntsepe, p 143.	I	1, 3, 5
	Fears over psychological reactions: depression, anxiety, suicide, “thinking too much” (1,2,3,5,6,19)	*“…for elder children some will show signs of fatigue with medication. You can hear a child asking why he must take another dose and he is not feeling sick.”* – Schenk p 24.		
	Health awareness /knowing ones status (1,3,4,5,6,11,17,18,22,23) Frustration surrounding side effects (9,11,12,14,16,19,21) Drug fatigue (7,12,14,16,18) Forgetting (8,12,18) Frustration surrounding lack of independence /reactance (9,17,18) Morning dose problems (oversleeping, late) (12,18) Travelling /not being home /school trips interfere with medication routine (8,9,11,18) Pill burden, complicated medication prep (14,18) Using alarms & reminder devices (11,14,18)	*“Especially if I am really busy with school or anything, I set an alarm so I can remember coz I easily forget to take my drugs.”* – Denison p 4.		
	Concerns over losing family support: financial or neglect/abuse (1,2,3,5)	*“After my father’s death we had a family meeting, where my family refused to let me stay with them.”* – Midtbo, p 267.		
	Guardian fails to give consent (3)			
	HIV+ family members as role models (3,10,20)	*“I had some fear inside: how will my parents accept it… If only I was above 18 years I could stand for myself but now I am dependent on my parents*.” Ferrand, p 2327.		
	Peer or guardian encouragement to test (3)			
	Empathy from family members (21)			
Family support *n* = 17	Full, timely disclosure by family (8,14,17,18,19,22,23)		F	1, 2, 4, 5
	Family assisted medication reminders (7,8,9,10,11,14,18,19,23)			
	Neglect /orphanhood (8,14,19) Punishment for adherence slips leads to dishonesty (16,18) Skills training, education and support for caregivers (11,20)	*“I: You said if your mother was still around things would not be happening the way they are now. Is there anything that you think would be happening differently..? R: Yes. I: Like what? R: Like love.” –* Peterson, p 972.		
	HIV counselling offered with no pressure to test (1,3,4,6)	*“Here (at the clinic) they may be talking about pills, but at the support group children will be talking about their experiences…” –* Mupambireyi p 109.		
	Pre- and post-test counselling available (3,6)			
	Community HIV & SRH educational available (2,19)			
Broader social support *n* = 17	ALHIV outreach services /peer support (19, 23)		C	1, 2, 3, 5
	School support /selective disclosure to “safe” teacher (6,10,14,18,23)			
	ALHIV community support groups (8,15,19,21,24) Sensitization of teachers & school staff (7,22) Treatment buddies (15,20) Testing at home /home based care (6,24)	*“When I was still in school I used to face cases of discrimination from other students, which lead me to tell my teacher to help me.” –* Midtbo, p 266.		
	Youth targeted services & hours (4,7,9,17,18,21,22,23)	*“At the moment, adolescents are being sidelined. Adults are able to talk for themselves and children are also represented but no one represents the adolescents”* Mburu, p 15	S	1, 2, 3, 5
Adolescent specific health services *n* = 12	Nurses, counselorscounsellors with similar characteristics to patient (age range, gender) (2,17) HCW trained to work with youth /ALHIV (18,20,24) HC facilatiesfacilities that offer HIV and SRH education (17,23) Clinic co-ordinated disclosure process with families (17,19) Full disclosure by HCWs of HIV status and what ARVs are for (15,23)	*“… they are at such a difficult stage in their lives, to add to that ‘Now you’ve got HIV’, and all the implications of that, it slays them.”* – Cluver, p S62.		
	Uninformed about services cost & location (1,2,3,4,6)	*“I would go to a clinic far from home. There are so many people attending the local clinic who know me. I wouldn’t want them to know.” –* Francis, p 338.		
	Uninformed /fearful of what testing proceedureprocedure entails (1,2,6) No privacy /confidentiality concerns (4)	*“People come and do things so fast because they want to go back fast – they talk fast, write drugs, and then say ‘Next’.”* – Mburu, p 15.		
Adequacy of health systems *n* = 12	Appointments take too long (waits /queues) (1,3,6,21) ReferedReferred/linked to care following diagnosis (21,24) HCWs sensitized to work with HIV+ people (21,24) Long distance to clinic (2,8) Support groups /mental health services offered at the clinic (11,12,14,19) Understaffed clinics (21)	*“At the clinic we try to be as friendly as we can with adolescents… but sometimes we have a lot of pressure, especially with adult clients, so you lose your temper.” –* Hodgson, p 1208.	S	1, 2, 3, 5
	Long term symptomatic /history of severe illness prompts testing (3)	*“You try and remind them [the child] how they were … Then they will say ‘I was not going to school’, ‘I was not able to do this and that’ and you will ask them if they want to go back to the same situation.” –* Bernays, p 277.		
Past illness narratives *n* = 10	HCW using reminders of past illness to motivate ART adherence (17) Individual’s ART adherence motivated by fear of becoming sick again (9,10,11,12,16,19,23) Motivation to adhere to ART falters after symptom recovery (9,12,16,18)	*“I took the ART for some months and I felt fine, so I stopped taking them because I felt I was no longer ill.” –* Midtbo, p 265.	I, S	1, 5
	Unable to meet indirect costs of care (travel to clinic, medication for opportunistic infections) (2,8,10,15,17,19)	*“I wanted to go on my own before I became seriously ill but I had no money for transport and to pay at the clinic.”* – Ferrand, p 2328.		
Financial instability *n* = 10	Incentives provided; food, soap, travel reimbursement, skills training (20)	*“Please government, give people [who] have HIV food. And more money.”* – Li, p 755.	F,S	2, 3, 4, 5
	Mandatory ART counselling not offered for free (22) Household food insecurity limits ART adherence (8,13,16,19)	*“I have a prescription to go and buy drugs [for OI treatment] for my child but I don’t have the money. This child needs medication immediately.”* – Schenk, p 21.		
	Non-full disclosure by guardians about what medication is for (11,12,13,15,23)	*“Then they say I have malaria… so they will give me medicine… but I cannot start taking the medicine if I do not know where I got the malaria from.”* – Mupambireyi, p 109.		
Social coping mechanisms *n* = 9	Assigning reasons for taking medication to alternate illness to prevent unintended disclosure (7,11,14,19,22)	*“… my friends don’t know that I had the virus [so I said] I felt some malaria.”* – Denison, p 2.	I,F	5
	Family goals /women planning pregnancy (1,3,4) Goals (family, career or education) facilitates linkage & retention in HIV care (10,19,23)	*“As a mother, I need to be healthy for the sake of my child. I want to set an example by making good decisions.”* – Francis, p 336.		
Future orientation *n* = 9	Perception of having “no future” following diagnosis as a barrier to testing and care enrollmentenrolment (2,4,10,20) Family, education or life goals as ART adherence motivators (10,12)	*“I am scared because I want to finish school and you know, get a job and have a boyfriend, but I don’t know how to do that as a person with HIV.” –* Li, p 755.	I	1, 2, 5
Media influence *n* = 7	Media influenced adolescents HIV knowledge /“Know Your Status” campaign (1,2,3,4,5,6)	*“I would go for VCT because it’s importland to know my status. It will help me stay protected and also protect others.”* – Francis, p 336.	C	1, 2
	Media campaigns to reduce HIV service stigma (2,22)			
	Traditional medicine used to avoid costs of care (7)			
Reliance on traditional medicine *n* = 5	Caregiver decision to treat with TM (12,13) TM practicionerspractitioners claiming to heal HIV (7,11,18) Medication mythology /mistrust of ART (12,18)	*“I used those herbs and I have even drunk urine because there is someone who came and lied to us that drinking urine can heal HIV.” –* Kunapareddy, p 391.	F,C	1, 2, 5

SEM levels: I – Individual; F – Family /Peer; C – Community; S – Structural.

Cascade stages: 1 – diagnosis; 2 – linkage to care; 3 – engagement /retention in care; 4 – ART initiation and adherence; 5 – viral suppression.

### Stigma

Debilitating experiences with stigma were described in every study, manifesting as internalized, anticipated and externalized barriers to progression through all stages of the cascade. Anticipated stigma (fears surrounding social exclusion) was common at the individual level, negatively influencing motivations to obtain an HIV diagnosis [39–46] and to follow up on test results [44,45]. This was reinforced by experiences with external stigma at other levels including: family neglect or ostracism from peers [39,40,43,47–50], community gossip [41–43,46], discrimination in school [49,51–54] or lack of empathy from health care workers (HCW) [45,50,55–57]. Such social conditions reinforced barriers to HIV care linkage, retention in HIV services and adherence; with ALHIV often choosing to prioritize secrecy around their disease status over unintended disclosure [48,51–55,57–60]. This choice frequently accounted for missed ART doses when social conditions were unfavourable, such as having minimal privacy in the home [49,51,53,57] or at school [49,52].

### Self-efficacy to adapt to a life with HIV

Adolescents understood that the event of receiving an HIV positive diagnosis would require them to make lifestyle changes that, in many instances, they lacked confidence to adopt. Fear surrounding the ability to manage the consequences of a positive diagnosis frequently created a barrier to testing [39,40,42,43,47]. Such diminished self-efficacy similarly affected ALHIVs’ capacity to maintain new health behaviours over time, such as the ART adherence necessary to achieve viral suppression, due to frustration surrounding the ongoing nature of treatment or side effects [48–53,57,58,60,61]. Lack of self-efficacy was often associated with an absence of empathetic support to adolescents offered within HIV services regarding how to integrate these required new behaviours into their lifestyles [45,49–53,57,58,60]. Self-efficacy was restored through acts or interventions that operated at other levels of the SEM including support from family members, participation in community support groups or support from treatment buddies [48–52,54,57–59].

### Family support

The presence or absence of family support strongly influenced adolescents’ testing, linkage to care, and adherence to ART. Definitions of “family” were broad, and could include biological parents, distant relatives, close friends and /or other caregivers. “Support” was defined as empathy, encouragement or care provided during sickness [52,57], having an HIV-positive role model [47,59] and assistance with medication reminders [48–52,54,57–59]. Lack of family support included non-full disclosure to ALHIV of their status [46,48–50,54,57,61], neglect or abuse [48–50] and failure to provide consent for services [47], all of which negatively influenced movement through the care cascade.

### Broader social support

The inclusion of social services in communities was cited as facilitating engagement at all stages of the HIV care cascade. ALHIV support groups [44,45,48,54,56], home-based care [43,62] and HIV /sexual and reproductive health education through school or outreach programmes [40,45,50,54] were all detailed as beneficial resources. The presence or lack of support within schools in particular was influential in terms of ART adherence, experiences with discrimination from other students or teachers negatively influenced the ability of ALHIV to carry medication and manage a dosing schedule at school [49,51,53,54], and crowded environments with no privacy, particularly in boarding schools, exacerbated fears surrounding unintended disclosure [49,52,57,58]. However, when selective disclosure to a “safe” teacher or school nurse was obtained, it frequently facilitated good adherence to ART [49,51,54,59].

### Adolescent-specific health services

ALHIV often faced difficulties in transitioning between paediatric and adult models of care; several studies reported that health services which catered specifically to adolescents’ needs positively influenced their progression through the cascade. Services which accommodated “youth-friendly” hours that didn’t interfere with school or work were often cited as facilitating access to HIV testing, pre-ART care and ART [41,45,46,51,57,58,61], as were HCWs who had undergone specific training to work with adolescents [44–46,57,62]. In addition, adolescents expressed a preference for HIV counsellors who shared similar characteristics to themselves (age range or gender) to avoid feeling that counselling messages were condescending [40,46]. Sexual and reproductive health education incorporated into health services [46] and HCW participation in the disclosure processes [46,50,54,56] were found to positively influence both ALHIV retention in care and adherence.

### Adequacy of health systems

Adolescents’ linkage to and retention in care were affected by the ability of the health system to provide efficient, confidential and comfortable services. This included the presence or absence of outreach efforts to inform ALHIV about service availability [39–41,43,47], pre- and post-test counselling options [39,41,43,47] and employment of sufficient numbers of HCW of various cadre [39,43,45,47]. The infrastructural capacity to avail space within health facilities to ensure privacy [45,62] and the total time required for appointments based on queues and travel to and from the clinic further compounded barriers to both HIV diagnosis and retention in care [39,40,43,45,47,48].

### Past illness narratives

Experiences with physical deterioration and severe illness could often facilitate engagement in HIV care, specifically in regard to receiving an HIV diagnosis [47] and adhering to treatment [50,52–54,58–60]. Within families and at health facilities, these past illness narratives were used as reminders by caregivers and HCWs to facilitate ALHIV’s adherence to ART [46,60]. Conversely, experiencing improvements in symptoms could also hinder adolescents’ ART adherence, particularly if their HIV status had not been fully disclosed to them, or they had limited knowledge about their illness and its treatment requirements: several studies identified faltering adherence among ALHIV who had regained physical strength and a “normal” appearance [51,53,57,58,60].

### Financial instability

The direct and indirect costs of care and treatment acted as a barrier to HIV care engagement, demonstrated at the family and structural level of the SEM. The cost of transportation to reach appointments [40,46,48,55,56,59] or additional food required to satisfy the nutritional needs of adolescents on ART [48,50,55,60] negatively influenced family willingness or ability to support ALHIV. At the structural level, the absence of available financial assistance to cover the costs of medications for opportunistic infections or ART counselling exacerbated the situation [50,60,61]. Conversely, one study found that the inclusion of economic incentives including food, household provisions or travel reimbursement encouraged linkage to care among ALHIV [44].

### Social coping mechanisms

In multiple studies, adolescents described a variety of coping mechanisms to navigate social situations that threatened unwanted disclosure. Such strategies included ALHIV claiming that their drugs, AIDS symptoms or prolonged school absences were the result of an alternate condition, such as heart problems or malaria [49,50,52–56,61]. These strategies were also used by caregivers and HCWs to avoid disclosure to younger ALHIV of their true condition at the family and structural SEM levels [51,54,57,58]. Such tactics were utilized to facilitate short-term ART adherence, despite their apparently limited effectiveness to help ALHIV achieve sustained adherence to treatment.

### Future orientation

Future aspirations, and the ability to identify personal goals including marriage, pregnancy or further education were cited in several studies as factors that motivated ALHIV to obtain an HIV diagnosis [39,41,47], to link to HIV care [58] and to adherence to their antiretroviral therapy [53,59]. However, adolescents’ fear of having diminished options in the future following a positive HIV diagnosis acted as a barrier to diagnosis and ART adherence [40,41,44,59]

### Media influence

Mass media campaigns were identified as effective in promoting knowledge surrounding HIV risk factors and diagnosis. The HIV testing campaign slogan ALHIV repeatedly referenced was the “Know Your Status” (KYS) campaign, that originated in Lesotho [63]. The “KYS” slogan and associated statements regarding the moral imperative of testing were recited by adolescents in multiple countries during interviews [39,41–43,47]. However, researchers noted that despite this familiarity with the KYS campaign, rates of testing often remained low [40,41]. Media campaigns were also detailed as a means of counteracting community stigma towards ALHIV that undermined HIV service enrolment [40,61].

### Reliance on traditional medicine

The use of traditional medicine was identified in the SEM at the family and community levels as a barrier to linkage to HIV care and to ART adherence. Reliance on traditional medicine for health care delayed the initiation of ART [52,57,60], and was favoured at times by caregivers [53,55] with reduced costs cited as justification for their use by economically strained families [60]. Lack of community HIV and ART education also contributed to the perpetuation of local disease mythologies associated with the use of traditional medicine to treat HIV [57,60].

## Discussion

Our meta-ethnographic approach to the analysis of qualitative literature published across varying contexts in SSA revealed striking thematic similarities across different countries and over time. Stigma, family support, self-efficacy and social support were common themes resonating across multiple settings in SSA and across the 10-year period of inquiry. The degree of consistency in these findings across diverse settings suggests that similar interventions to improve the uptake of HIV care among ALHIV may be relevant in many SSA countries.

Out of 24 selected articles, only seven investigated multiple stages of the HIV care cascade. Most of the studies focused on discreet aspects of HIV care and treatment, in particular testing or adherence, leaving stages such as linkage to care and initiation onto treatment under-represented. In order to better understand the impacts of efforts being made to achieve the UNAIDS 90-90-90 targets [64], further investigation of the full spectrum of ALHIV experiences with HIV care is needed.

During the time-period evaluated, the most significant efforts to promote HIV engagement have primarily acted at the structural level: for example, free ART provision, expansion of counselling and testing services and formation of support groups in many clinics. However, it was the threat of social isolation as a result of attitudes towards HIV that emerged as the most influential factor hindering ALHIV progression through all stages of the care cascade. Detrimental experiences with stigma were the most salient theme across the entire time-span, suggesting that structural-level interventions have had limited impact on individual experiences of living with HIV over the last 10 years.

Given that structural interventions alone are unlikely to act at multiple levels of the SEM, the potential of youth-specific services to improve rates of care retention in SSA (a facilitator to HIV care engagement detailed in numerous articles) is ultimately reliant on the presence of mutually improved conditions at the other SEM levels [65]. Their ability to facilitate the movement of ALHIV through the stages of the care cascade is contingent upon caregiver support, community perceptions of services, and experiences of stigma associated with their use. Further, training new or existing HCWs to work with adolescents, expanding services to youth-friendly hours and improving upon the efficiency of appointments all require a sustained and substantial economic commitment [66] which is in turn dependent on international funding priorities and domestic health policies [67–70].

We noted that stigma was commonly anticipated by ALHIV, often manifest through fear of unwanted disclosure of their positive status after being seen using HIV services or taking medication. These individual level fears pervaded into other SEM levels, where they threatened to deteriorate relationships with loved ones or result in ostracism by the community. The power of such anticipated stigma to negatively affect adolescent care engagement in SSA cannot be overstated, particularly as it compounds the hardships that many ALHIV have already gone through following early parental loss and learning their own positive HIV status. This appears distinct from the dominant manifestations of stigma amongst adults living with HIV in SSA [71,72]. The stigma experienced by many adults living with HIV is inextricably linked to blame or promiscuity [51,73]. Witnessing such enacted stigma demonstrated towards adults may not only reinforce anticipated stigma in ALHIV at the individual level, but also presents a barrier to their care at other levels of the SEM where they require the support of adults. For example, guardian experiences’ with enacted stigma may result in failure to provide ALHIV with the consent and accompaniment required to access HIV care in some areas, out of their own unwillingness to be seen utilizing HIV services [51,74,75]. Our findings suggest that interventions to address stigma need to account for the inherent differences between ALHIV and adults living with HIV. Care should be taken to not assume that the same forces driving stigma amongst an adult population resonate amongst adolescents.

In the absence of coordinated support to address treatment challenges among ALHIV, motivational techniques were often applied by caregivers or HCWs, such as the use of past illness narratives to facilitate ART adherence based on ALHIVs’ desires to maintain “normalcy” [49,53,57,58,60]. Our meta-ethnography highlights that HCW used the motivation of “staying well” to encourage adherence, insinuating a comparison to a past period of ill health. Further, both ALHIV and their caregivers reported inventing an alternate illness to attribute medication use to, either to avoid disclosure from caregiver to ALHIV or from ALHIV to the wider community of extended family, peers and teachers. The use of past or alternative illness narratives represent makeshift approaches to adherence and disclosure that have been utilized in the absence of broader social support to help ALHIV manage care dilemmas attached to stigma. Recent changes in guidance for ART eligibility to include all who test HIV positive regardless of their immune status or WHO staging [69,76] should reduce the occurrence of “past illnesses” and could therefore change how ALHIV engage with HIV care. Social approaches to managing engagement in HIV care and adherence should encourage early and ongoing disclosure assisted by HCWs, rather than being left to the responsibility of families alone [30,77–79]. This coordinated disclosure will need to place greater emphasis on sustainable facilitators to self-efficacy, such as future orientation, to avoid use of short-term tactics that fail to recognize the capacity of ALHIV to understand and manage their illness.

The self-efficacy of ALHIV is recognized as a key facilitator to diagnosis, retention in care and adherence to ART. However, its effect is reliant on social conditions such as family environment, presence or absence of peer support and the attitudes of HCWs working with youth. Inclusion of supportive social conditions at different levels of the SEM, such as community support groups, family counselling and HIV /sexual and reproductive health education, has the potential to bolster self-efficacy and motivate ALHIV through the stages of care [80–83]. In the absence of supportive conditions, ALHIV self-efficacy may fail to develop or result in rebellion against their care management [84] as they gain further independence with age, yet maintain a sense of social isolation and frustration surrounding treatment adaptation.

**Figure 4.**
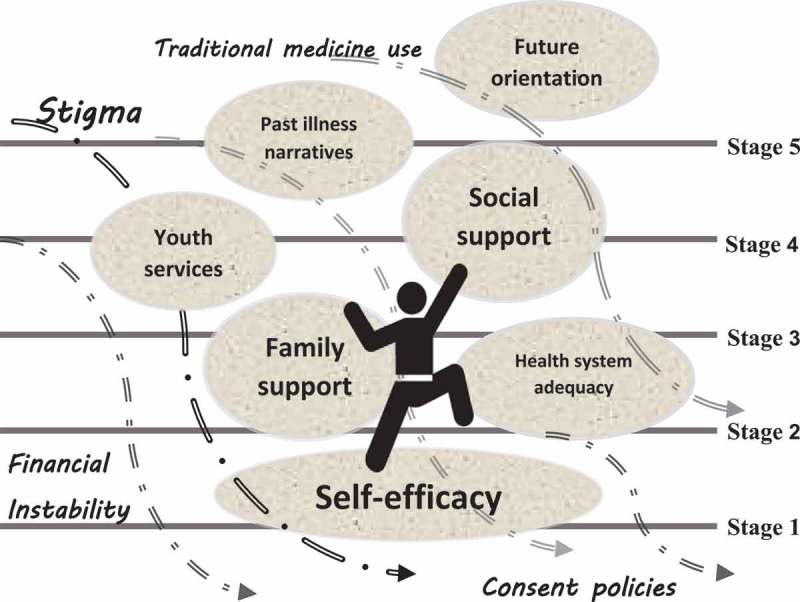
“Scaling a waterfall”: upward momentum among ALHIV through the HIV care cascade stages.

We concur with others that have taken a critical stance on the utility of a linear model of the HIV care cascade with a proposed end-point of viral suppression [85]. Our systematic review of evidence suggests that such a model is not sufficient to generalize the ongoing and evolving process of managing a lifelong condition for ALHIV. In Figure 4, we illustrate how the reality is a succession of ebbs and flows as ALHIV attempt to “scale a waterfall” to achieve viral suppression. Ethnographic and qualitative descriptions portray movement which requires upward momentum to move through these stages, with ALHIV using facilitators as footholds to manoeuvre against a downward cascade of barriers that persistently threaten to undermine their intentions to engage in HIV care.

Our study is affected by various limitations inherent to qualitative studies and the meta-ethnography design. Adolescents may be prone to social desirability bias in their responses, particularly when discussing behaviours such as ART adherence or clinic attendance. In addition, most of the studies in our analysis adopted a cross-sectional rather than a longitudinal design, which may have been better suited to identify issues related to transition between paediatric and adult models of HIV care. Our search was limited to publications in English which may partially explain the geographic gaps in the research studies that we identified; however, these gaps may also be influenced by differences in research funding across countries. Although our search criteria were broad, some relevant studies may not have been captured by our choice of terms; yet this risk should be minimized by our strategy of searching through the reference lists of relevant articles. Furthermore, due to limited available research exploring gendered differences in adolescent HIV care, our ability to unpick gender-specific factors influencing cascade progression was limited. Additional qualitative research should be undertaken to explore the risk perceptions, knowledge of HIV transmission, sources of SRH education and experiences of adolescent females with HIV services, not only because they are at higher risk of acquiring HIV [6,64,86,87], but also because their experiences with stigma may profoundly reinforce barriers to testing or ART for PMTCT [80,88,89].

To our knowledge, this is the first meta-ethnography of research on factors influencing engagement in HIV care among adolescents in SSA that examines treatment progression holistically, by synthesizing studies focused on different aspects of the care cascade. Our use of a socio-ecological lens provides a comprehensive view of the issues influencing HIV care engagement, and enables us to analyse the interplay between individual, family, community and structural factors. The findings generated through this review are timely given the current focus on adolescent health within global health initiatives and also since the adolescent HIV epidemic in the region has peaked, and readiness to test, commence treatment and adhere successfully to ART is essential in reducing transmission rates and preventing drug resistance [77]. Significant efforts are still required to ensure that this important age group do not fall further behind in the global effort to eradicate AIDS by 2030 [90].

## Conclusions

For ALHIV in SSA, individual-level issues are the most influential in preventing engagement across all stages of the HIV care cascade, although family, community and structural issues also play an important role. Despite scale-up of services and increased availability of ART in SSA over the past decade, stigma remains the most pervasive barrier to HIV care engagement, present at each level of the SEM, and hindering progression across all stages of the care cascade. Psychosocial interventions among adolescents and their families should be prioritized to improve adolescents’ engagement in HIV care, enhance their wellbeing and ultimately reduce their risk of ill-health and mortality.

## References

[1] UNICEF For every child, end AIDS – seventh stocktaking report. New York; 2016.

[2] WHO |Joint United Nations Programme on HIV/AIDS. Treating 3 million by 2005: making it happen: the WHO strategy. WHO: Geneva; 2003.

[3] WHO Prevention of HIV in infants and young children: review of evidence and WHO’s activities. WHO: Geneva; 2002.

[4] Lowenthal ED, Bakeera-Kitaka S, Marukutira T, Chapman J, Goldrath K, Ferrand RA. Perinatally acquired HIV infection in adolescents from sub-Saharan Africa: a review of emerging challenges. Lancet Infect Dis. 2014;14(7):627–17. 24406145 10.1016/S1473-3099(13)70363-3 PMC4074242

[5] Sohn AH, Hazra R The changing epidemiology of the global paediatric HIV epidemic: keeping track of perinatally HIV-infected adolescents. J Int AIDS Soc. 2013;16:1–8. 23782474 10.7448/IAS.16.1.18555 PMC3687075

[6] Idele P, Gillespie A, Porth T, Suzuki C, Mahy M, Kasedde S, et al Epidemiology of HIV and AIDS among adolescents: current status, inequities, and data gaps. J Acquir Immune Defic Syndr. 2014;66(SUPPL. 2):SS144–53. 10.1097/QAI.0000000000000176 24918590

[7] Patton GC, Sawyer SM, Santelli JS, Ross DA, Afifi R, Allen NB, et al Our future: a Lancet commission on adolescent health and wellbeing. Lancet. 2016;387(10036):2423–78. 27174304 10.1016/S0140-6736(16)00579-1 PMC5832967

[8] Fatti G, Shaikh N, Eley B, Grimwood A Improved virological suppression in children on antiretroviral treatment receiving community-based adherence support: a multicentre cohort study from South Africa. AIDS Care. 2014;26(4):448–53. 24215157 10.1080/09540121.2013.855699

[9] Ferrand RA, Corbett EL, Wood R, Hargrove J, Ndhlovu CE, Cowan FM, et al AIDS among older children and adolescents in Southern Africa: projecting the time course and magnitude of the epidemic. AIDS. 2009;23(15):2039–46. 19684508 10.1097/QAD.0b013e32833016ce PMC3408596

[10] Doyle AM, Mavedzenge SN, Plummer ML, Ross DA The sexual behaviour of adolescents in sub-Saharan Africa: patterns and trends from national surveys. Trop Med Int Health. 17;2012:796–807. 22594660 10.1111/j.1365-3156.2012.03005.x

[11] Evans D, Menezes C, Mahomed K, Macdonald P, Untiedt S, Levin L, et al Treatment outcomes of HIV-infected adolescents attending public-sector HIV clinics across Gauteng and Mpumalanga, South Africa. AIDS Res Hum Retroviruses. 2013;29(6):892–900. 23373540 10.1089/aid.2012.0215 PMC3653371

[12] Dow DE, Shayo AM, Cunningham CK, Reddy EA Durability of antiretroviral therapy and predictors of virologic failure among perinatally HIV-infected children in Tanzania: a four-year follow-up. BMC Infect Dis. 2014;14:567. 25373425 10.1186/s12879-014-0567-3 PMC4225040

[13] Zanoni BC, Archary M, Buchan S, Katz IT, Haberer JE Systematic review and meta-analysis of the adolescent HIV continuum of care in South Africa: the cresting wave. BMJ Glob Heal. 2016;1(3):e000004. 10.1136/bmjgh-2015-000004 PMC5321340 28588949

[14] Kimani-Murage EW, Manderson L, Norris SA, Kahn K “It’s my secret”: barriers to paediatric HIV treatment in a poor rural South African setting. AIDS Care-Psychol Socio-Med Asp AIDS/HIV. 2013;25(6):744–47. 10.1080/09540121.2012.748865 PMC3756619 23244783

[15] Pound P, Britten N, Morgan M, Yardley L, Pope C, Daker-White G, et al Resisting medicine: a synthesis of qualitative studies of medicine taking. Soc Sci Med. 2005;61(1):133–55. 15847968 10.1016/j.socscimed.2004.11.063

[16] BMJ Qualitative research methodologies: ethnography. BMJ. 2008;337:a1020. 18687725 10.1136/bmj.a1020

[17] Merten S, Kenter E, McKenzie O, Musheke M, Ntalasha H, Martin-Hilber A Patient-reported barriers and drivers of adherence to antiretrovirals in sub-Saharan Africa: a meta-ethnography. Trop Med Int Heal. 2010;15(s1):16–33. 10.1111/j.1365-3156.2010.02510.x 20586957

[18] Musheke M, Ntalasha H, Gari S, Mckenzie O, Bond V, Martin-Hilber A, et al A systematic review of qualitative findings on factors enabling and deterring uptake of HIV testing in sub-Saharan Africa. BMC Public Health. 2013;220:13. 23497196 10.1186/1471-2458-13-220 PMC3610106

[19] Noblit GW, Hare RDR Meta-ethnography: synthesizing qualitative studies. Vol. 11, Qualitative Research Methods. Newbury Park: Sage Publications; 1988 p. 88.

[20] Britten N, Campbell R, Pope C, Donovan J, Morgan M, Pill R Using meta ethnography to synthesise qualitative research: a worked example. J Health Serv Res Policy. 2002;7(4):209–15. 12425780 10.1258/135581902320432732

[21] Zimmer L Qualitative meta-synthesis: a question of dialoguing with texts. J Adv Nurs. 2006;53:311–18. 16441536 10.1111/j.1365-2648.2006.03721.x

[22] Centers for Disease Control and Prevention (CDC) Understanding the HIV care continuum [Internet]. 2016 Available from: https://www.cdc.gov/hiv/pdf/library/factsheets/cdc-hiv-care-continuum.pdf

[23] Office of HIV/AIDS and Infectious Disease Policy (OHAIDP), US Dept of Health and Human Services (HHS) HIV /AIDS Care Continuum [Internet]. Washington (DC); 2017 Available from: https://www.aids.gov/federal-resources/policies/care-continuum/

[24] Ferrand RA, Briggs D, Ferguson J, Penazzato M, Armstrong A, MacPherson P, et al Viral suppression in adolescents on antiretroviral treatment: review of the literature and critical appraisal of methodological challenges. Trop Med Int Heal. 2016;21(3):325–33. 10.1111/tmi.12656 PMC4776345 26681359

[25] Kim S-H, Gerver SM, Fidler S, Ward H Adherence to antiretroviral therapy in adolescents living with HIV: systematic review and meta-analysis. AIDS. 2014;28(13):1945–56. 24845154 10.1097/QAD.0000000000000316 PMC4162330

[26] MacPherson P, Munthali C, Ferguson J, Armstrong A, Kranzer K, Ferrand RA, et al Service delivery interventions to improve adolescents’ linkage, retention and adherence to antiretroviral therapy and HIV care. Trop Med Int Heal. 2015;20(8):1015–32. 10.1111/tmi.12517 PMC4579546 25877007

[27] Varga C, Brookes H Factors influencing teen mothers’ enrollment and participation in prevention of mother-to-child HIV transmission services in Limpopo Province, South Africa. Qual Health Res. 2008;18(6):786–802. 18503020 10.1177/1049732308318449

[28] Psaros C, Remmert JE, Bangsberg DR, Safren SA, Smit JA Adherence to HIV care after pregnancy among women in sub-Saharan Africa: falling off the cliff of the treatment cascade. Curr HIV /AIDS Rep. 2015;12(1):1–5. 25620530 10.1007/s11904-014-0252-6 PMC4370783

[29] Shroufi A, Ndebele W, Nyathi M, Gunguwo H, Dixon M, Saint-Sauveur JF, et al Risk of death among those awaiting treatment for HIV infection in Zimbabwe: adolescents are at particular risk. J Int AIDS Soc. 2015;18(1):19247. 25712590 10.7448/IAS.18.1.19247 PMC4339242

[30] Aderomilehin O, Hanciles-Amu A, Ozoya OO Perspectives and practice of HIV disclosure to children and adolescents by health-care providers and caregivers in sub-Saharan Africa : a systematic review. Front Public Heal. 2016;4:166. 10.3389/fpubh.2016.00166 PMC4981616 27570762

[31] Kaufman MR, Cornish F, Zimmerman RS, Johnson BT Health behavior change models for HIV prevention and AIDS care: practical recommendations for a multi-level approach. J Acquir Immune Defic Syndr. 2014;66(Suupl 3):S250–S258. 25007194 10.1097/QAI.0000000000000236 PMC4536982

[32] Hosek SG, Harper GW, Lemos D, Martinez J An ecological model of stressors experienced by youth newly diagnosed with HIV. J HIV/AIDS Prev Youth Child. 2008;9(2):192–218. 10.1080/15538340902824118 PMC2834206 20216916

[33] Mugavero MJ, Norton WE, Saag MS Health care system and policy factors influencing engagement in HIV medical care: piecing together the fragments of a fractured health care delivery system. Clin Infect Dis. 2011;52(supp 2):S238–46. 21342913 10.1093/cid/ciq048 PMC3106258

[34] Busza J, Walker D, Hairston A, Gable A, Pitter C, Lee S, et al Community-based approaches for prevention of mother to child transmission in resource-poor settings: a social ecological review. J Int AIDS Soc. 2012;15(Suppl 2):17373. 22789640 10.7448/IAS.15.4.17373 PMC3499910

[35] Gourlay A, Birdthistle I, Mburu G, Iorpenda K, Wringe A Barriers and facilitating factors to the uptake of antiretroviral drugs for prevention of mother-to-child transmission of HIV in sub-Saharan Africa: a systematic review. J Int AIDS Soc. 2013;16:18588. 23870277 10.7448/IAS.16.1.18588 PMC3717402

[36] Dixon-Woods M, Shaw RL, Agarwal S, Smith JA The problem of appraising qualitative research. Qual Saf Health Care. 2004;13(3):223–25. 15175495 10.1136/qshc.2003.008714 PMC1743851

[37] Validity LL reliability, and generalizability in qualitative research. J Fam Med Prim Care. 2015;4(3):324–47. 10.4103/2249-4863.161306 PMC4535087 26288766

[38] Better Value Healthcare Ltd Critical Appraisal Skills Programme (CASP) [Internet]. 2013 Available from: http://www.casp-uk.net/

[39] Råssjö E-B, Darj E, Konde-Lule J, Olsson P Responses to VCT for HIV among young people in Kampala, Uganda. Ajar-African J AIDS Res. 2007;6(3):215–22. 10.2989/16085900709490417 25866167

[40] Izugbara CO, Undie-C-C, Mudege NN, Ezeh AC Male youth and voluntary counseling and HIV-testing: the case of Malawi and Uganda. Sex Educ. 2009;9(3):243–59.

[41] Francis D “They should know where they stand”: attitudes to HIV voluntary counselling and testing amongst a group of out-of-school youth. South African J Educ. 2010;30(3):327–42.

[42] Ntsepe Y, Simbayi LC, Shisana O, Rehle T, Mabaso M, Ncitakalo N, et al Perceptions about the acceptability and prevalence of HIV testing and factors influencing them in different communities in South Africa. Sahara J-J Soc Asp HIV-AIDS. 2014;11(1):138–47. 10.1080/17290376.2014.937355 PMC4272100 25059467

[43] Strauss M, Rhodes B, George G A qualitative analysis of the barriers and facilitators of HIV counselling and testing perceived by adolescents in South Africa. BMC Heal Serv Res. 2015;15:250. 10.1186/s12913-015-0922-0 PMC4484707 26123133

[44] Petersen I, Bhana A, Myeza N, Alicea S, John S, Holst H, et al Psychosocial challenges and protective influences for socio-emotional coping of HIV+ adolescents in South Africa: a qualitative investigation. AIDS Care. 2010;22(8):970–78. 20229370 10.1080/09540121003623693 PMC3037257

[45] Hodgson I, Ross J, Haamujompa C, Gitau-Mburu D Living as an adolescent with HIV in Zambia - lived experiences, sexual health and reproductive needs. AIDS Care - Psychol Socio-Medical Asp AIDS/HIV. 2012;24(10):1204–10. 10.1080/09540121.2012.658755 22380932

[46] Cluver LD, Hodes RJ, Toska E, Kidia KK, Orkin FM, Sherr L, et al “HIV is like a tsotsi. ARVs are your guns”: associations between HIV-disclosure and adherence to antiretroviral treatment among adolescents in South Africa. AIDS. 2015;29(Suppl 1):S57–S65. 26049539 10.1097/QAD.0000000000000695

[47] Ferrand RA, Trigg C, Bandason T, Ndhlovu CE, Mungofa S, Nathoo K, et al Perception of risk of vertically acquired HIV infection and acceptability of provider-initiated testing and counseling among adolescents in Zimbabwe. Am J Public Health. 2011;101(12):2325–32. 22021300 10.2105/AJPH.2011.300250 PMC3222430

[48] Mavhu W, Berwick J, Chirawu P, Makamba M, Copas A, Dirawo J, et al Enhancing psychosocial support for HIV positive adolescents in Harare, Zimbabwe. PLoS One. 2013;8(7):e70254. 23894625 10.1371/journal.pone.0070254 PMC3720910

[49] Mutumba M, Bauermeister JA, Musiime V, Byaruhanga J, Francis K, Snow RC, et al Psychosocial challenges and strategies for coping with HIV among adolescents in Uganda: a qualitative study. AIDS Patient Care STDS. 2015;29(2):86–94. 25607900 10.1089/apc.2014.0222

[50] Mattes D “Life is not a rehearsal, it’s a performance”: an ethnographic enquiry into the subjectivities of children and adolescents living with antiretroviral treatment in northeastern Tanzania. Child Youth Serv Rev. 2014;45:28–37.

[51] Schenk KD, Kiragu K, Murugi J, Sarna A If you build it, will they come? A qualitative investigation into community barriers to accessing paediatric HIV services in Kenya. Child Youth Serv Rev. 2014;45(C):18–27.

[52] Denison JA, Banda H, Dennis AC, Packer C, Nyambe N, Stalter RM, et al “The sky is the limit”: adhering to antiretroviral therapy and HIV self-management from the perspectives of adolescents living with HIV and their adult caregivers. J Int AIDS Soc. 2015;18:19358. 25591915 10.7448/IAS.18.1.19358 PMC4296051

[53] Nyogea D, Mtenga S, Henning L, Franzeck FC, Glass TR, Letang E, et al Determinants of antiretroviral adherence among HIV positive children and teenagers in rural Tanzania: a mixed methods study. BMC Infect Dis. 2015;15:28. 25637106 10.1186/s12879-015-0753-y PMC4314748

[54] Midtbø V, Shirima V, Skovdal M, Daniel M, Smith DK, Toledo L, et al How disclosure and antiretroviral therapy help HIV-infected adolescents in sub-Saharan Africa cope with stigma. Afr J AIDS Res. 2012;11(3):261–71. 25860100 10.2989/16085906.2012.734987

[55] Vreeman RC, Nyandiko WM, Ayaya SO, Walumbe EG, Inui TS Cognitive interviewing for cross-cultural adaptation of pediatric antiretroviral therapy adherence measurement items. Int J Behav Med. 2014;21(1):186–96. 23188670 10.1007/s12529-012-9283-9

[56] Mupambireyi Z, Bernays S, Bwakura-Dangarembizi M, Cowan FM “I don’t feel shy because I will be among others who are just like me … ”: the role of support groups for children perinatally infected with HIV in Zimbabwe. Child Youth Serv Rev. 2014;45:106–13. 25284920 10.1016/j.childyouth.2014.03.026 PMC4167251

[57] Kunapareddy CJ, Nyandiko W, Inui T, Ayaya S, Marrero DG, Vreeman R A qualitative assessment of barriers to antiretroviral therapy adherence among adolescents in Western Kenya. J HIV AIDS Soc Serv. 2014;13(4):383–401. 28367106 10.1080/15381501.2012.754392 PMC5374741

[58] Lowenthal ED, Marukutira TC, Chapman J, Mokete K, Riva K, Tshume O, et al Psychosocial assessments for HIV plus African adolescents: establishing construct validity and exploring under-appreciated correlates of adherence. PLoS One. 2014;9(10):e109302. 25279938 10.1371/journal.pone.0109302 PMC4184864

[59] Li RJ, Jaspan HB, O’Brien V, Rabie H, Cotton MF, Nattrass N Positive futures: a qualitative study on the needs of adolescents on antiretroviral therapy in South Africa. AIDS Care - Psychological and Socio-Medical Aspects of AIDS/HIV.2010;22(6):758. 10.1080/09540120903431363 20467942

[60] Bernays S, Seeley J, Rhodes T, Mupambireyi Z What am I “living” with? Growing up with HIV in Uganda and Zimbabwe. Sociol Health Illn. 2015;37(2):270–83. 25421409 10.1111/1467-9566.12189

[61] Mburu G, Ram M, Oxenham D, Haamujompa C, Iorpenda K, Ferguson L Responding to adolescents living with HIV in Zambia: a social-ecological approach. Child Youth Serv Rev. 2014;45:9–17.

[62] Busza J, Besana GVR, Mapunda P, Oliveras E Meeting the needs of adolescents living with HIV through home based care: lessons learned from Tanzania. Child Youth Serv Rev. 2014;45:137–42.

[63] WHO Regional Office for Africa Know your status HIV campaign receives praise for the achievements accomplished in Lesotho. [Internet]. World Health Organization: Maseru; 2008 Available from: http://www.afro.who.int/en/lesotho/press-materials/item/323-know-your-status-hiv-campaign-receives-praise-for-the-achievements-accomplished-in-lesotho.html

[64] UNAIDS 90-90-90: an ambitious treatment target to help end the AIDS epidemic [Internet]. 2014 Available from: http://www.unaids.org/Sites/Default/Files/Media_Asset/90-90-90_En_0.Pdf

[65] Skovdal M, Belton S The social determinants of health as they relate to children and youth growing up with HIV infection in sub-Saharan Africa. Child Youth Serv Rev. 2014;45:1–8.

[66] Remme M, Siapka M, Sterck O, Ncube M, Watts C, Vassall A Financing the HIV response in sub-Saharan Africa from domestic sources: moving beyond a normative approach. Soc Sci Med. 2016;169:66–76. 27693973 10.1016/j.socscimed.2016.09.027

[67] Belton S, Skovdal M “Getting to Zero ”: the policy role of social determinants of health as they relate to children and youth living with HIV in sub-Saharan Africa. Child Youth Serv Rev. 2014;45(2014):160–63.

[68] Mark D, Taing L, Cluver L, Collins C, Iorpenda K, Andrade C, et al What is it going to take to move youth-related HIV programme policies into practice in Africa? J Int AIDS Soc. 2017;20(Suppl 3):112–16. 10.7448/IAS.20.4.21491 PMC5577735 28530047

[69] World Health Organization Consolidated guidelines on the use of antiretroviral drugs for treating and preventing HIV infection: recommendations for a public health approach. WHO: 2nd Geneva; 2016. 27466667

[70] Nattrass N, Hodes R, Cluver L Changing donor funding and the challenges of integrated HIV treatment. AMA J Ethics. 2016;18(7):681–90. 27437818 10.1001/journalofethics.2016.18.7.ecas3-1607

[71] Campbell C, Foulis CA, Maimane S, Sybia Z “I have an evil child at my house”: stigma and HIV/AIDS management in a South African community. Am J Public Health. 2005;95(5):808–15. 15855456 10.2105/AJPH.2003.037499 PMC1449259

[72] Pantelic M, Boyes M, Cluver L, Thabeng M “ They say HIV is a punishment from God or from ancestors ”: cross-cultural adaptation and psychometric assessment of an HIV stigma scale for South African adolescents living with HIV (ALHIV-SS). Child Indic Res. 2016. 10.1007/s12187-016-9428-5 PMC5816760 29497463

[73] Roura M, Urassa M, Busza J, Mbata D, Wringe A, Zaba B Scaling up stigma? The effects of antiretroviral roll-out on stigma and HIV testing. Early evidence from rural Tanzania. Sex Transm Infect. 2009;85(4):308–12. 19036776 10.1136/sti.2008.033183 PMC2708343

[74] Pitorak H, Bergmann H, Fullem A, Duffy M Mapping HIV services and policies for adolescents: a survey of 10 countries in sub-Saharan Africa. Arlington (VA): AIDSTAR-One; 2013 Available from: https://aidsfree.usaid.gov/sites/default/files/aidstar-one_report_mapping_hiv_services.pdf

[75] Busza J, Strode A, Dauya E, Ferrand RA Falling through the gaps: how should HIV programmes respond to families that persistently deny treatment to children? J Int AIDS Soc. 2016;19(1):20789. 27221666 10.7448/IAS.19.1.20789 PMC4879178

[76] WHO 4.1 Preparing people living with HIV for ART [Internet]. Geneva: WHO; 2016 Available from: http://www.who.int/hiv/pub/arv/chapter4.pdf?ua=1

[77] WHO HIV and adolescents: guidance for HIV testing and counseling and care for adolescents living with HIV: recommendations for a public health approach and considerations for policy-makers and managers [Internet]. Geneva: WHO; 2013 Available from: http://apps.who.int/iris/bitstream/10665/94334/1/9789241506168_eng.pdf?ua=1 25032477

[78] Mutumba M, Musiime V, Tsai AC, Byaruhanga J, Kiweewa F, Bauermeister JA, et al Disclosure of HIV status to perinatally infected adolescents in urban Uganda: a qualitative study on timing, process, and outcomes. J Assoc Nurses AIDS Care. 2015;26(4):472–84. 26066697 10.1016/j.jana.2015.02.001

[79] World Health Organization Guideline on HIV disclosure counselling for children up to 12 years of age [Internet]. Geneva: WHO; 2011 Available from: http://www.who.int/hiv/pub/hiv_disclosure/en/ 26158185

[80] Levy ME, Ong’wen P, Lyon ME, Cohen CR, D’Angelo LJ, Kwena Z, et al Low social support and HIV-related stigma are highly correlated among adolescents living with HIV in Western Kenya. J Adolesc Heal. 2016;58(2):S82.

[81] Vu L, Burnett-Zieman B, Banura C, Okal J, Elang M, Ampwera R, et al Increasing uptake of HIV, sexually transmitted infection, and family planning services, and reducing HIV-related risk behaviors among youth living with HIV in Uganda. J Adolesc Heal. 2017;60(2):S22–8. 10.1016/j.jadohealth.2016.09.007 28109336

[82] Woollett N, Cluver L, Hatcher A, Brahmbhatt H To be HIV positive is not the end of the world ” : resilience among perinatally infected HIV positive adolescents in Johannesburg. Child Youth Serv Rev. 2016;70:269–75.

[83] Fournier B, Bridge A, Kennedy AP, Alibhai A Hear our voices: a photovoice project with children who are orphaned and living with HIV in a Ugandan group home. Child Youth Serv Rev. 2014;45:55–63.

[84] Adejumo OA, Malee KM, Ryscavage P, Hunter SJ, Taiwo BO Contemporary issues on the epidemiology and antiretroviral adherence of HIV-infected adolescents in sub-Saharan Africa: a narrative review. J Int AIDS Soc. 2015;18:20049. 26385853 10.7448/IAS.18.1.20049 PMC4575412

[85] Paparini S, Rhodes T The biopolitics of engagement and the HIV cascade of care: a synthesis of the literature on patient citizenship and antiretroviral therapy. Crit Public Health. 2016;26(5):501–17.

[86] Dellar RC, Dlamini S, Karim QA Adolescent girls and young women: key populations for HIV epidemic control. J Int AIDS Soc. 2015;18:64–70. 10.7448/IAS.18.2.19408 PMC4344544 25724504

[87] Stöckl H, Kalra N, Jacobi J, Watts C Is early sexual debut a risk factor for HIV infection among women in sub-Saharan Africa? A systematic review. Am J Reprod Immunol. 2013;69(Suppl 1):27–40. 23176109 10.1111/aji.12043

[88] Forrest JI, Kaida A, Dietrich J, Miller CL, Hogg RS, Gray G Perceptions of HIV and fertility among adolescents in Soweto, South Africa: stigma and social barriers continue to hinder progress. AIDS Behav. 2009;13:S55–61. 10.1007/s10461-009-9552-z 19343491

[89] Chandra-Mouli V, Armstrong A, Amin A, Ferguson J A pressing need to respond to the needs and sexual and reproductive health problems of adolescent girls living with HIV in low- and middle-income countries. J Int AIDS Soc. 2015;18(Suppl 5):20297. 26643463 10.7448/IAS.18.6.20297 PMC4672396

[90] United Nations General Assembly Transforming our world: the 2030 agenda for sustainable development. In: A/RES/70/1. 2015 p. 35.

